# Untreated dental caries in Brazilian adolescents: analysis by the PUFA index

**DOI:** 10.1590/1980-549720260010.supl.1

**Published:** 2026-07-06

**Authors:** Lívia Natália Sales Brito, Gustavo Gomes Agripino, Sandra Aparecida Marinho, Helene Soares Moura, Robeci Alves Macêdo, Pierre Andrade Pereira de Oliveira

**Affiliations:** I Universidade Estadual da Paraíba – Araruna (PB), Brazil.

**Keywords:** Oral health, Dental caries, Adolescent health, Dental health surveys

## Abstract

**Objective:**

To describe the distribution profile of untreated dental caries in Brazilian adolescents, using the PUFA index, and identify the main associated factors.

**Methods:**

This is a cross-sectional study based on data from the 2023 National Survey of Oral Health. The sample consisted of 8,053 adolescents aged 15 to 19 years. The dependent variable was the presence of untreated caries, measured by the PUFA index. Independent variables included sociodemographic and oral health-related factors such as impacts on quality of life and access to dental services. Descriptive and bivariate analyses were performed using the Rao-Scott test and multiple logistic regression, considering a significance level of 5% (p<0.05).

**Results:**

We verified that 12.5% of Brazilian adolescents had at least one tooth affected by some repercussion of untreated caries. There were statistically significant associations between PUFA≥1 and the adolescents’ socioeconomic conditions, such as lower level of education, low family income, being beneficiary of government cash transfer programs, in addition to Black skin color/race. We observed a negative impact on the quality of life of 22.4% of adolescents with PUFA index ≥1, and 19.1% had never had a dental appointment. Low family income, presence of pain in the last six months, and self-perceived need for dental treatment were factors strongly associated with PUFA≥1.

**Conclusion:**

Social determinants influenced the progression of dental caries in adolescents. Therefore, social policies targeting Black adolescents and expanded access to dental services are essential to reduce oral health problems in this more vulnerable population.

## INTRODUCTION

Oral problems are increasingly recognized as an integral part of general health, with physical, emotional, and psychological repercussions^1,2^. In this context, over the last decades, governments and nongovernmental organizations have intensified efforts to promote the oral health of the population. According to data from the World Health Organization (WHO), the prevalence of dental caries has declined in several regions worldwide^3^.

The National Oral Health Policy (*Política Nacional de Saúde Bucal* – PNSB) has been implemented since 2004, known as *Brasil Sorridente* [Smiling Brazil], guiding the organization of oral health actions within the context of the Brazilian Unified Health System (SUS). In 2023, its consolidation as a State Policy represented a milestone for the sustainability of these actions, by ensuring financing and institutional integration. Within this context, national epidemiological surveys, such as the Brazilian Oral Health Project (SB Brasil), assume strategic roles in providing systematic and representative evidence on the oral health conditions of the population, subsidizing the planning, monitoring, and evaluation of actions, strengthening oral health surveillance and contributing to the reduction of inequalities^4^.

Dental caries can be influenced by different social determinants, and management strategies should include prevention plans and age-based systemic plans, as this factor plays a relevant role^5^. Evidence on the impact of pulp impairment due to caries on the quality of life of children and adolescents may raise public health managers’ awareness of the importance of preventive actions, early diagnosis, and treatment of dental caries^2^.

The PUFA index has been used to evaluate the presence of oral conditions due to untreated dental caries, by recording the progression of the dental caries to the surrounding tissues, including pulp involvement (P), ulceration (U), fistula (F), and abscess (A). Thus, four clinical stages of advanced dental caries are classified by the PUFA, showing prevalent oral conditions relevant to the planning of health actions^6^. This index reflects the pulp and periapical involvement underlying the carious lesion and serves as a screening instrument for the indication of complementary examinations and diagnostic determination^7^.

In 2023, the evaluation by the PUFA index was incorporated into the National Survey of Oral Health (SB Brasil), allowing to estimate the severity of the disease and identify associated pathologies^8^. Therefore, the development and implementation of effective strategies for the control of dental caries, especially in the context of social inequalities and their impact on the younger population, became extremely relevant and urgent^9^. Thus, understanding the distribution of untreated pulp conditions, from the perspective of public health, allows evaluating the treatment needs more accurately and guiding the adequate allocation of resources for dental care.

In the present study, we aimed to describe the distribution profile of untreated dental caries in adolescents, based on the PUFA index as well as to identify the main factors associated with these diseases.

## METHODS

This is a cross-sectional study based on secondary data from SB Brasil (2023). This is an epidemiological survey of national scope, linked to the PNSB, in which the oral health conditions of the Brazilian population is investigated. The adopted sampling process was probabilistic, stratified and by multiple-stage clusters. Additional details on the methodological design are found in the technical publications of SB Brasil 2023^8,10^.

The sample analyzed in the present study included 8,053 adolescents, aged between 15 and 19 years, who were interviewed and examined at their homes.

The dependent variable was the presence of at least one lesion due to untreated dental caries with clinical repercussion, according to the PUFA index. The variable was dichotomized in PUFA≥1 (presence of one or more lesions) and PUFA=0 (absence of lesions).

Independent variables included sociodemographic aspects (sex, age, self-reported skin color/race, level of education, family income, beneficiary of government cash transfer programs, and region), in addition to factors related to oral health (pain in the last six months, self-reported need for treatment, loss of permanent teeth, bleeding gums, dental calculus, malocclusion, and perceived urgency of treatment). Variables related to oral health and its impacts on quality of life, as well as the use of dental services, were also included.

For the analyses, the complex sampling plan of SB Brasil 2023 was considered, which includes stratification, clusters (primary sampling units), and sample weights adjusted to the probability of selection. To ensure representative estimates and correct standard errors, the complex sample plan file was applied using the stratum components, primary sampling unit, and weight provided by the survey.

Statistical analyses were performed using the Statistical Package for the Social Sciences (SPSS) software, version 20, with application of the complex sampling module. Initially, descriptive analysis was carried out and then bivariate analyses were performed to test associations between PUFA≥1 and independent variables, using the Rao-Scott test, adjusted version of the χ^2^ test for complex samplings. Finally, a multiple logistic regression analysis was conducted, including variables with p<0.05 in the bivariate analysis. After the regression analysis, only the independent variables that remained with p<0.05 after adjustment were kept in the final model. The final model estimated the crude and adjusted odds ratios (OR), with their respective 95% confidence intervals (95%CI), adopting a significance level of 5% (p<0.05).

The multicollinearity between the independent variables presented in the final model was evaluated by tolerance, variance inflation factor (VIF), and condition indices, and no indicative values of collinearity (tolerance 0.90–0.97; VIF 1.02–1.11; higher condition index=14.95) were observed. Thus, all variables were kept in the multivariate model.

There was no need for submission to the Research Ethics Committee because it is a study based on secondary data. SB Brasil 2023 was previously approved by the National Commission of Ethics in Research — CONEP (Certificate of Presentation for Ethical Consideration — CAAE No. 34497120.6.3001.0008; Opinion No. 4.823.054).

### Data Availability Statement

The entire dataset supporting the results of this study is available in the very article.

## RESULTS

The sample included 8,053 adolescents, aged between 15 and 19 years, of whom 12.5% presented the PUFA index ≥1, that is, at least one tooth affected by untreated carious lesion, with the most common occurrence being pulp involvement (Table 1).

**Table 1:** Prevalence of untreated dental caries and oral lesions in permanent teeth in adolescents in Brazil, SB Brasil 2023.

Indicator	Present			Absent		
	%	95%CI	n	%	95%CI	n
**PUFA≥1**	12.5	10.1–15.2	1,076	87.5	84.8–89.9	6,965
**Pulp involvement**	9.9	7.9–12.3	848	90.1	87.7–92.1	7,193
**Ulceration**	2.2	1.7–3.0	251	97.8	97.0–98.3	7,790
**Fistula**	1.7	0.9–2.9	100	98.3	97.1–99.1	7,941
**Abscess**	0.4	0.3–0.7	47	99.6	99.3–99.7	7,994

Percentages calculated based on the weighted data of the sample plan of SB Brasil; n reflects the crude value. PUFA≥1: presence of at least one permanent tooth with pulp involvement, fistula, or abscess; 95%CI: 95% confidence interval.

When considering the PUFA index ≥1 and the demographic characteristics of the evaluated population, we identified associations with some socioeconomic characteristics, such as: skin color/race, low level of education, family income, and beneficiaries of cash transfer programs, as detailed in Table 2.

**Table 2:** Prevalence of PUFA≥1 in permanent teeth according to sociodemographic characteristics in Brazilian adolescents, SB Brasil 2023.

Variable	Category	Sample % (n)	PUFA≥1 prevalence (%)	95%CI	F	p-value
**Sex**	Boys	50.2 (3,871)	12.8	9.3–17.3	0.098	0.754
	Girls	49.8 (4,170)	12.1	10.0–14.7		
**Age (years)**	15–16	43.2 (3,868)	12.7	10.1–15.9	0.225	0.792
	17–18	39.6 (2,749)	11.8	8.7–15.7		
	19	17.2 (1,424)	13.5	9.0–19.7		
**Skin color/race**	White	38.0 (2,600)	6.8	5.0–9.3	8.538	<0.001^*^
	Black	12.5 (1,026)	18.4	13.1–25.3		
	Asian	1.3 (107)	11.1	5.2–22.3		
	Mixed-race	47.9 (4,199)	15.4	12.1–19.4		
	Indigenous	0.4 (19)	27.9	5.3–72.9		
**Level of Education**	Illiterate/Youth and Adult Education	0.5 (49)	42.2	16.0–73.7	10.957	<0.001^*^
	Elementary School	22.7 (2,000)	19.8	15.5–25.0		
	High School	71.5 (5,431)	10.4	7.8–13.6		
	Higher Education	5.4 (479)	5.1	2.5–10.2		
**Family income (MW)**	Up to 1	30.7 (1,472)	18.6	13.5–25.1	19.017	<0.001^*^
	>1 to 3	49.0 (2,514)	13.5	10.5–17.2		
	>3	20.3 (1,025)	2.9	1.7–4.9		
**Beneficiary of *Bolsa Família***	Yes	32.9 (2,579)	20.4	15.4–26.4	24.782	<0.001^*^
	No	67.1 (4,783)	8.6	6.7–11.0		
**Receives BPC**	Yes	6.8 (518)	24.4	15.4–36.3	10.137	0.001^*^
	No	93.2 (6,790)	11.6	9.3–14.2		
**Region**	North	10.8 (2,006)	19.4	14.9–24.9	2.240	0.094
	Northeast	29.5 (2,859)	13.3	9.9–17.7		
	Southeast	38.2 (919)	10.6	6.4–17.2		
	South	13.3 (1,088)	8.6	5.4–13.5		
	Midwest	8.3 (1,181)	15.0	12.2–18.4		

PUFA≥1: presence of at least one permanent tooth with pulp involvement, fistula, or abscess; 95%CI: 95% confidence interval; MW: minimum wage; *Bolsa Família*: cash transfer program from the Brazilian government; BPC: Continuous Cash Benefit from the Brazilian government; Adjusted F: test for independence with Rao-Scott correction for complex samples; Sample: absolute (unweighted) number of individuals by category;

*p<0.05: statistically significant.

We used the Oral Impacts on Daily Performances (OIDP) instrument to evaluate the impact of oral problems on adolescents’ quality of life. The instrument allowed us to observe that, among those adolescents whose oral health impacted their quality of life, 22.4% had a PUFA index ≥1. The associations between PUFA≥1 and the variables related to daily activities were significant, in which adolescents reported: stop playing sports (50.5%), stop having fun (43.0%), having impairments in daily activities (42.8%), sleeping poorly (35.9%), getting nervous/angry (34.1%), difficulty speaking (31.6%) or eating (31.3%), and being ashamed when smiling or talking (21.6%), as highlighted in Table 3.

**Table 3:** Association between impact on quality of life (OIDP) and presence of PUFA≥1 in permanent teeth of Brazilian adolescents, SB Brasil 2023.

Variable (OIDP)	Category	PUFA≥1 (%)	PUFA=0 (%)	95%CI PUFA≥1	Adjusted F	p-value
**OIDP (some impact)**	Yes	22.4	77.6	17.7–27.9	66.596	<0.001^*^
	No	6.4	93.6	4.9–8.3		
**Difficulty eating**	Yes	31.3	68.7	24.9–38.5	98.089	<0.001^*^
	No	8.0	92.0	6.3–10.2		
**Difficulty speaking**	Yes	31.6	68.4	19.1–47.5	17.116	<0.001^*^
	No	11.2	88.8	9.2–13.6		
**Discomfort when brushing**	Yes	14.1	85.9	8.7–22.2	0.385	0.535
	No	12.1	87.9	9.8–14.9		
**Stopped playing sports**	Yes	50.5	49.5	38.8–62.1	102.953	<0.001^*^
	No	11.1	88.9	9.0–13.7		
**Got nervous or angry**	Yes	34.1	65.9	26.1–43.1	87.938	<0.001^*^
	No	8.3	91.7	6.7–10.1		
**Stopped going out/having fun**	Yes	43.0	57.0	31.5–55.3	71.680	<0.001^*^
	No	10.3	89.7	8.3–12.7		
**Felt ashamed when smiling or talking**	Yes	21.6	78.4	15.5–29.3	17.755	<0.001^*^
	No	10.4	89.6	8.4–12.9		
**It affected daily activities**	Yes	42.8	57.2	28.0–59.1	36.819	<0.001^*^
	No	10.3	89.7	8.5–12.6		
**Slept poorly due to oral problems**	Yes	35.9	64.1	28.6–43.9	155.151	<0.001^*^
	No	7.3	92.7	5.9–9.2		

PUFA≥1: presence of at least one permanent tooth with pulp involvement, fistula, or abscess; 95%CI: 95% confidence interval (for PUFA≥1); Adjusted F: test for independence with Rao-Scott correction for complex samples;

*p<0.05: statistically significant; OIDP: Oral Impacts on Daily Performances.

In Table 4, we show the harms associated with the presence of PUFA index ≥1. Thus, it is worth noting that 17.5% of adolescents identified the need for treatment through self-assessment. In turn, 79.5% of adolescents with PUFA≥1 had a self-perception of immediate emergency treatment, 30.5% of these adolescents reported pain in the last six months, and 26.1% reported loss of at least one permanent tooth.

**Table 4:** Association between oral health disorders and the use of dental services with the presence of PUFA≥1 in permanent teeth in Brazilian adolescents, SB Brasil 2023.

Variable	Category	PUFA≥1 (%)	PUFA=0 (%)	95%CI PUFA≥1	Adjusted F	p-value
**Pain in the last 6 months**	Yes	30.5	69.5	24.2–37.6	132.479	<0.001^*^
	No	7.1	92.9	5.7–8.8		
**Self-perception of need for treatment**	Yes	17.5	82.5	14.4–21.2	79.171	<0.001^*^
	No	2.8	97.2	1.7–4.4		
**Perceived urgency of treatment**	Immediate	79.5	20.5	68.1–87.6	86.207	<0.001^*^
	Elective	14.8	85.2	11.3–19.2		
	Preventive/Routine	2.7	97.3	1.5–4.7		
	No need	2.7	97.3	0.9–7.4		
**Loss of permanent teeth (≥1)**	Yes	26.1	73.9	19.5–34.0	27.799	<0.001^*^
	No	10.3	89.7	8.1–13.0		
**Malocclusion**	Present	16.0	84.0	12.6–20.1	5.124	0.024^*^
	Absent	10.7	89.3	8.1–14.1		
**Bleeding gums**	Present	17.8	82.2	14.5–21.6	14.555	<0.001^*^
	Absent	9.7	90.3	7.1–13.0		
**Presence of dental calculus**	Present	16.5	83.5	13.4–20.1	8.130	0.004^*^
	Absent	10.2	89.8	7.5–13.8		
**Had never had a dental appointment**	Had never had it	19.1	80.9	12.6–27.9	5.185	0.023^*^
	Had already had it	11.5	88.5	9.5–14.0		
**Last dental appointment (year)**	≤1	11.2	88.8	8.9–14.1	0.205	0.651
	>1	12.0	88.0	9.3–15.3		
**Type of service sought**	SUS	17.8	82.2	13.7–22.8	36.088	<0.001^*^
	Private/Health insurance	6.0	94.0	4.5–8.0		
**Type of service in the last appointment**	SUS	16.8	83.2	13.5–20.7	35.557	<0.001^*^
	Private/Health insurance	6.7	93.3	5.1–8.8		

PUFA≥1: presence of at least one permanent tooth with pulp involvement, fistula, or abscess; 95%CI: 95% confidence interval (PUFA≥1); SUS: Brazilian Unified Health System; Adjusted F: test for independence with Rao-Scott correction for complex samples;

*p<0.05: statistically significant.

Moreover, we noticed that, among adolescents with PUFA≥1, 19.1% had never had a dental appointment. However, 32.0% of adolescents seeking dental care reported tooth ache, and 17.7% of adolescents sought care by the SUS, according to Table 4.

In the logistic regression model, we observed that the main factors associated with the presence of PUFA≥1 were socioeconomic inequalities and the presence of oral health problems. After adjusting the model, adolescents of Black skin color/race were 2.41 times more likely to present PUFA≥1 compared to white teenagers. In addition, those belonging to families with income up to one minimum wage presented 3.22 times higher odds of PUFA≥1 than adolescents with income above three minimum wages. Presence of pain (OR=3.54), self-reported need for treatment (OR=3.59), and tooth loss (OR=1.881) were also factors strongly associated with the presence of PUFA≥1, after adjusting the odds, according to Table 5.

**Table 5:** Logistic regression model between sociodemographic factors and oral health problems with PUFA≥1 in Brazilian adolescents, SB Brasil 2023.

Variable	Comparative category	OR	95%CI	p-value	Adjusted OR	95%CI (adjusted)	p-value
		Crude analysis			Adjusted analysis		
**Race/skin color**	Black vs. White	3.07	1.85–5.09	<0.001^*^	2.41	1.27–4.56	0.030^*^
	Mixed-race vs. White	2.47	1.75–3.49		1.39	0.88–2.19	
**Level of Education**	Incomplete Elementary School vs. Higher Education	4.24	1.92–9.40	<0.001^*^	2.30	0.78–6.71	0.005^*^
	Complete Elementary School vs. Higher Education	5.69	2.28–14.19		1.99	0.65–6.07	
	High School vs. Higher Education	2.15	0.98–4.72		1.10	0.40–3.02	
**Family income (MW)**	Up to 1 vs. >3	7.68	3.97–14.85	<0.001^*^	3.22	1.69–6.14	0.007^*^
	1 to 3 vs. >3	5.23	2.89–9.45		3.08	1.68–5.67	
**Pain in the last 6 months**	Pain vs. No pain	5.75	4.16–7.94	<0.001^*^	3.54	2.47–5.08	<0.001^*^
**Self-perception of need for treatment**	Yes vs. No	7.44	4.48–12.37	<0.001^*^	3.59	1.94–6.66	<0.001^*^
**Tooth loss (≥1 tooth)**	Yes vs. No	3.07	1.99–4.74	<0.001^*^	1.88	1.06–3.33	0.031^*^

OR: odds ratio; 95%CI: 95% confidence interval;

*p<0.05: statistically significant; MW: minimum wage.

## DISCUSSION

In the present study, we evaluated data on 8,053 Brazilian adolescents aged between 15 and 19 years, an age group in which physical, emotional, and social changes make oral appearance and functionality relevant to self-esteem and quality of life.

As it is a cross-sectional study, we cannot establish the time frame of the observed associations, as there is a risk of reverse causality. Hence, we cannot state whether the clinical conditions preceded the impacts or whether they influenced the perception and behavior in oral health. Thus, the verified associations should be interpreted with caution.

It is worth highlighting the frequency of 12.5% of Brazilian adolescents aged between 15 and 19 years presenting PUFA index ≥1, that is, they have at least one tooth affected by untreated carious lesion, having pulp involvement as the main consequence (9.9%). Likewise, Gomes *et al.* ^9^, when assessing adolescents aged 12 years, observed that the clinical consequences of untreated carious lesions (PUFA index ≥1) were verified in 8.71% of the sample, with pulp involvement also being the most frequent. This is particularly worrisome due to the severity of carious lesions and their consequences, especially when considering the small interval (three years) between the ages of adoles-cents evaluated in both studies, with an increase in the frequency of those affected by almost four (3.99) percentage points, considering the age group between 15 and 16 years (12.7%). Furthermore, in the present study, although not significant, there was an increase in the prevalence of the PUFA index ≥1 among the age groups of 17–18 years, ranging from 11.8 to 13.5% at 19 at years of age, with worse oral conditions at this age. Handling this situation in public health is complex and requires intersectoral management of oral health services, with planning, resource management, and greater investments in primary and specialized care to identify and treat teeth with pulp involvement^9^.

The prevention of dental caries and its proper treatment go beyond the functional and aesthetic dimensions of teeth. Oral health transcends the oral environment, influencing the general health and quality of life of the individual^11^. In addition, oral conditions during childhood may affect adulthood^12^. The clinical consequences of untreated dental caries were associated with sleep problems in school-age children^13^. This fact was corroborated by the present study, as we verified a statistically significant association between PUFA index ≥1 and the quality of sleep (35.9%) of adolescents. These results emphasize the importance of immediate care in the face of the consequences of dental caries and represent a crucial indicator for the establishment of priorities in public health services and for clinical decision-making^14^.

The type of food consumed is also related to the higher prevalence of caries in early ages. Through a systematic review, Cascaes *et al.* ^15^ observed that the intake of ultra-processed foods was associated with greater occurrence of dental caries in childhood and adolescence, evidencing the need for public policies aimed at modifying social determinants and reducing the consumption of these foods, aiming to improve oral health of children and adolescents^2,15,16^.

The unequal distribution of dental caries among populations is strongly associated with socioeconomic status^17,18^. In the present study, socioeconomic characteristics, such as skin color/race, level of education, family income, and beneficiary of cash transfer programs, were statistically associated with PUFA index ≥1. However, many variables have been considered predictors of dental caries throughout adolescence. In the SB Brasil 2010, living in cities without fluoridated water, with lower water supply coverage, and in municipalities with lower median income were considered strong determinants for dental caries. In addition, skin color, low family income, and presence of periodontal clinical conditions, such as dental calculus or bleeding gums, were associated with individual determinants of dental caries in children aged 12 years^5,17,19^.

When assessing the prevalence and severity of caries in 12-year-old Brazilian children and their association with individual and contextual factors, Freire *et al.* ^17^ identified that Black and mixed-race children had a significantly higher prevalence of dental caries than white children. However, the authors pointed out that this difference was mostly due to worst socioeconomic conditions of Black and mixed-race skin color strata in the Brazilian population. These pieces of evidence can be extrapolated to the results of the present study, considering that, in addition to the Black and mixed-race adolescent population, most of the Indigenous population also presented PUFA index ≥1, all populations of low socioeconomic conditions. Moreover, adolescents in families with lower socioeconomic status, as well as those belonging to families with family cohesion (affective bond among its members) from low to moderate, were more likely to present a greater number of cavitated carious lesions^5,20^, corroborating the findings of the present study, in which PUFA≥1 was three times more prevalent in individuals with income below three minimum wages, even after adjusting the variables.

Higher levels of education and income of the parents are associated with greater attention and care for the oral health of children^21,22^. Maternal education may be associated with greater search for dental services among adolescents, as parental choices still have an impact on the decisions of individuals in this period^23^. However, studies whose authors evaluate adolescents’ level of education and the PUFA index are still scarce in the literature, indicating the importance of the findings of this research, considering that a statistically significant association was identified between lower level of education of Brazilian adolescents and PUFA index ≥1. Inequalities persist, with higher prevalence of dental caries in poorer groups, reflecting the striking social inequalities in Brazil and their impacts on the population’s health^17^.

Thus, we evidenced the need for an integral public health policy that articulates family, school, and health services. The scope of services should be increasingly expanded, as socioeconomic disparities are still a limiting factor in access to dental services. Furthermore, the context in which adolescents are inserted should also be considered. Awareness of early oral care should involve the family and schools, so that strategies are proposed to reduce the risk of oral diseases in childhood and adolescence. Adolescents from unfavorable school environments (students from public schools and with a higher rate of grade retention) were more likely to seek dental services for treatment and resolution of symptoms^23^ of pain resulting from the already deteriorated dental condition, which was observed in the present study by the self-perception of immediate urgent treatment in the vast majority (79.5%) of adolescents with PUFA≥1. The great demand for SUS care by these adolescents is highlighted.

Adolescence is a transition period from childhood to adulthood, involving psychological changes and strong social influences, as well as greater exposure to emotional, social, and physical risk factors, becoming a phase vulnerable to harmful and complex behaviors^18,24^. In this context, the OIDP is inserted as an instrument that measures the perception of individuals about the social impact of oral disorders on their well-being. Authors of different studies have identified correlations between the presence of dental caries and the reduction in the quality of life of adolescents by using this instrument^1,25^. Such findings corroborate the ones identified in the present study, considering the association found between worst quality of life and presence of PUFA≥1 in permanent teeth of Brazilian adolescents. It is worth highlighting some of the factors associated with the reduction in their quality of life, as the consequences of untreated dental caries prevented these adolescents from playing sports (50.5%), having fun (43.0%) and performing daily activities (42.8%), in addition to affecting their sleep (35.9%). Such evidence on the impact of dental caries progression on the quality of life of children and adolescents subsidize greater attention of public policy makers regarding the provision of dental care based on prevention, early diagnosis, and treatment of carious lesions^2^.

In addition, the PUFA index allows us to trace pulp diseases through intraoral clinical signs; however, the index presents greater sensitivity when there is noticeable dental cavitation. In cases of restored teeth, this diagnostic sensitivity decreases^7^. As it is a chronic process, dental caries presents pulp signs and symptoms that vary according to the phase, acute or chronic, and a dental abscess may become asymptomatic after spontaneous fistula drainage. Thus, a single evaluation may not fully reflect the condition of a child with advanced dental caries^2^.

According to our results, we emphasize that there was a significant association between PUFA index ≥1 and the presence of health problems of adolescents such as the need for emergency care, loss of permanent teeth, malocclusion, and periodontal diseases. Therefore, adolescents with PUFA index ≥1 showed a higher probability of requiring invasive clinical interventions. Among these adolescents, 79.5% had a self-perception of immediate need for dental treatment in emergency situations. Ferreira *et al.* ^26^ pointed out that poor self-rated oral health by young people, or insufficient oral health appreciation, may increase the search for a dental surgeon only for symptom resolution, which is corroborated by the present study. Lopes *et al.* ^23^ highlighted that the probability of adolescents with cavitated lesions seeking dental services due to pain was 9.0% higher than that of those without cavitated lesions. This situation has implications for the health of the individual and impacts health services, which may present a higher demand for emergency care.

Moreover, we identified a significant association between the use of public dental services (17.7%) and the presence of PUFA≥1 in permanent teeth among Brazilian adolescents. Besides, 32.0% of adolescents who sought dental care reported tooth ache, reflecting the progression of caries and the late search for care, already with pulp involvement and pain. This reinforces the importance of adequate identification of more susceptible groups in order to better target and optimize disease control strategies and resources^2,9^.

Oral hygiene habits should be encouraged to reduce the incidence of dental caries^16^. There are an increasing number of risk factors that increase the likelihood of developing oral problems and dental caries. Nevertheless, it has not yet been determined which factors present the greatest risks^18^. Hence, an investigation into these risk factors in adolescents, particularly in developing countries, becomes paramount, as the results may vary depending on regional differences^20^.

In addition to family income and level of education, one of the main risk factors for the development of pulp tissue disorders due to untreated dental caries in adolescents was the skin color/race factor, considering that the odds of adolescents with Black skin color/race presenting PUFA≥1 was 2.41 times higher than that of white adolescents, regardless of their level of education or family income. The same was verified for mixed-race adolescents (OR 1.39), although in lower proportion. Nonetheless, Freire *et al.* ^17^ attributed this higher prevalence of dental caries to the lower family income of Black individuals. According to Vazquez *et al.* ^18^, the distribution of dental caries in populations is directly related to socioeconomic conditions. However, in the present study, we identified that, when level of education and income were included in the logistic regression model as the main socioeconomic factors, the skin color/race variable remained statistically significant for PUFA≥1.

The causes of oral health inequalities remain interrelated and complex. Early oral health interventions can positively influence the effectiveness of actions, especially among the most underprivileged groups^27^. The Family Health Strategy is fundamental for supporting Health Surveillance, by ensuring the monitoring of the user from the first contact to the continuity of care, enabling greater knowledge of the territory and the social context^28^.

This study contributes to the practice of dental surgeons in Primary Health Care by providing evidence for the identification of vulnerable groups and priority oral disorders, qualifying the planning of actions in the territory. By strengthening oral health surveillance, the findings subsidize the organization of the agenda, risk stratification, and targeting of prevention and care strategies, as well as supporting managers in defining priorities and rational allocation of resources, thus strengthening oral health actions in public services^8^.

Therefore, social policies focused on Black adolescents, aimed at expanding access to dental services, both from primary and specialized care, either through preventive or interventional actions, are paramount for reducing these oral health problems in this population with greater vulnerability.
