# Supplementary material for: Untreated dental caries in Brazilian adolescents: analysis by the PUFA index
Source: Rev Bras Epidemiol. 2026 Jul 6;29(Suppl 1):e260010.supl1. doi: 10.1590/1980-549720260010.supl.1 (PMC13336697; doi:10.1590/1980-549720260010.supl.1)
Supplement: Translation

# Translation: Cárie não tratada em adolescentes do Brasil: análise pelo índice PUFA

**DOI:** 10.1590/1980-549720260010.supl.1.2

**Authors:** Lívia Natália Sales Brito, Gustavo Gomes Agripino, Sandra Aparecida Marinho, Helene Soares Moura, Robeci Alves Macêdo, Pierre Andrade Pereira de Oliveira

**Affiliations:** I Universidade Estadual da Paraíba – Araruna (PB), Brasil.

**Keywords:** Saúde bucal, Cárie dentária, Saúde do adolescente, Inquéritos de saúde bucal

## Abstract

**Objetivo:**

Descrever o perfil de distribuição de cáries não tratadas em adolescentes brasileiros, utilizando o índice PUFA, identificando os principais fatores associados.

**Métodos:**

Trata-se de um estudo transversal, baseado em dados da Pesquisa Nacional de Saúde Bucal de 2023. A amostra foi composta de 8.053 adolescentes entre 15 e 19 anos. A variável dependente foi a presença de lesão de cárie não tratada, mensurada pelo índice PUFA. As variáveis independentes incluíram fatores sociodemográficos e relacionados à saúde bucal, como qualidade de vida e acesso aos serviços odontológicos. Foram realizadas análises descritivas e bivariadas, utilizandose os testes de Rao-Scott e de regressão logística múltipla, considerando-se nível de significância de 5% (p<0,05).

**Resultados:**

Foi verificado que 12,5% dos adolescentes apresentaram, ao menos, um dente acometido por alguma repercussão da cárie não tratada. Houve associações significativas entre o PUFA ≥1 e as condições socioeconômicas dos adolescentes, como menor escolaridade, renda familiar reduzida e participação em programas governamentais, além da cor/raça preta. Houve impacto negativo na qualidade de vida de 22,4% dos adolescentes com índice PUFA ≥1, sendo que 19,1% nunca haviam realizado consulta odontológica. Baixa renda familiar, presença de dor nos últimos seis meses e autopercepção de necessidade de tratamento odontológico foram fatores fortemente associados ao PUFA ≥1.

**Conclusão:**

Determinantes sociais influenciaram a progressão da doença cárie em adolescentes. Assim, políticas sociais direcionadas aos adolescentes de cor/raça preta e ampliação do acesso de serviços odontológicos são essenciais para a redução de agravos em saúde bucal nessa população de maior vulnerabilidade.

## INTRODUÇÃO

Os problemas bucais são cada vez mais reconhecidos como parte integrante da saúde geral, com repercussões físicas, emocionais e psicológicas^1,2^. Nesse contexto, ao longo das últimas décadas, governos e organizações não governamentais têm intensificado esforços para promover a saúde bucal da população. Dados da Organização Mundial da Saúde (OMS) mostram que a prevalência de cárie tem apresentado declínio em diversas regiões do mundo^3^.

A Política Nacional de Saúde Bucal (PNSB) vem sendo implementada desde 2004, sob a denominação Brasil Sorridente, orientando a organização das ações em saúde bucal no âmbito do Sistema Único de Saúde (SUS). Em 2023, sua consolidação como Política de Estado representou um marco para a sustentabilidade dessas ações, ao assegurar financiamento e integração institucional. Nesse cenário, os inquéritos epidemiológicos nacionais, como o Projeto Saúde Bucal Brasil (SB Brasil), assumem papéis estratégicos ao fornecer evidências sistemáticas e representativas sobre as condições de saúde bucal da população, subsidiando o planejamento, o monitoramento e a avaliação das ações, fortalecendo a vigilância em saúde bucal e contribuindo para a redução das desigualdades^4^.

A cárie dentária pode ser influenciada por diferentes determinantes sociais, e as estratégias de manejo devem incluir planos de prevenção e de assistência baseados em faixas etárias, por esse fator desempenhar um papel relevante^5^. Evidências sobre o impacto do comprometimento pulpar por cárie na qualidade de vida de crianças e adolescentes podem sensibilizar gestores de saúde pública quanto à importância de ações preventivas, do diagnóstico precoce e do tratamento da cárie dentária^2^.

O índice PUFA tem sido utilizado para avaliar a presença de condições bucais decorrentes de cáries não tratadas, por meio do registro da progressão da lesão cariosa para os tecidos circundantes, incluindo envolvimento pulpar (P), ulceração (U), fístula (F) e abscesso (A). Desse modo, o PUFA classifica quatro estágios clínicos da cárie avançada, evidenciando condições bucais prevalentes e relevantes para o planejamento de ações em saúde^6^. Esse índice reflete o envolvimento pulpar e periapical, subjacente à lesão cariosa, e serve como um instrumento de triagem para a indicação de exames complementares e a determinação diagnóstica^7^.

Em 2023, a avaliação por meio do índice PUFA foi incorporada à Pesquisa Nacional de Saúde Bucal (SB Brasil), possibilitando estimar a gravidade da doença e identificar patologias associadas^8^. Diante disso, a elaboração e a implementação de estratégias eficazes para o controle da cárie dentária, especialmente no contexto das desigualdades sociais e seu impacto sobre a população mais jovem, tornaram-se extremamente relevantes e urgentes^9^. Assim, compreender a distribuição das condições pulpares não tratadas, da perspectiva da saúde pública, permite avaliar com maior precisão as necessidades de tratamento e orientar a alocação adequada de recursos para o cuidado odontológico.

Destarte, o presente estudo teve como objetivo descrever o perfil de distribuição das cáries não tratadas em adolescentes, com base no índice PUFA, bem como identificar os principais fatores associados a esses agravos.

## MÉTODOS

Este é um estudo transversal, baseado em dados secundários provenientes do SB Brasil (2023). Trata-se de um inquérito epidemiológico de abrangência nacional que investiga as condições de saúde bucal da população brasileira e está vinculado à PNSB. O processo de amostragem adotado foi probabilístico, estratificado e por conglomerados em múltiplos estágios. Detalhes adicionais sobre o delineamento metodológico encontram-se nas publicações técnicas do SB Brasil 2023^8,10^.

A amostra analisada no presente estudo incluiu 8.053 adolescentes, com idades entre 15 e 19 anos, que foram entrevistados e examinados em seus domicílios.

A variável dependente foi a presença de pelo menos uma lesão decorrente de cárie não tratada com repercussão clínica, conforme o índice PUFA. A variável foi dicotomizada em PUFA ≥1 (presença de uma ou mais lesões) e PUFA=0 (ausência de lesões).

As variáveis independentes englobaram aspectos sociodemográficos (sexo, idade, cor/raça autodeclarada, escolaridade, renda familiar, recebimento de auxílio governamental e região), além de fatores relacionados à saúde bucal (dor nos últimos seis meses, autorrelato de necessidade de tratamento, perda de dentes permanentes, sangramento gengival, cálculo dental, oclusopatias e urgência percebida de tratamento). Também foram incluídas variáveis referentes à saúde bucal e aos seus impactos na qualidade de vida, bem como ao uso de serviços odontológicos.

Para as análises, foi considerado o plano amostral complexo do SB Brasil 2023, que inclui estratificação, conglomerados (unidades primárias de amostragem) e pesos amostrais ajustados à probabilidade de seleção. Para garantir estimativas representativas e erros padrão corretos, aplicou-se o arquivo com plano de amostra complexa, utilizando os componentes de estrato, unidade primária de amostragem e peso fornecidos pelo inquérito.

As análises estatísticas foram realizadas utilizando o *software Statistical Package for the Social Sciences* (SPSS), versão 20, com aplicação do módulo de amostragem complexa. Inicialmente, procedeu-se à análise descritiva e, em seguida, às análises bivariadas, para testar associações entre PUFA ≥1 e as variáveis independentes, utilizando-se o teste de Rao-Scott, versão ajustada do teste qui-quadrado para amostragens complexas. Por fim, foi conduzida uma análise de regressão logística múltipla, incluindo variáveis com p<0,05 na análise bivariada. Após a análise de regressão, foram mantidas no modelo final apenas as variáveis independentes que permaneceram com p<0,05 após o ajuste. O modelo final estimou os *odds ratios* (OR) brutos e ajustados, com seus respectivos intervalos de confiança de 95%, adotando-se nível de significância de 5% (p<0,05).

A multicolinearidade entre as variáveis independentes apresentadas no modelo final foi avaliada por tolerância, fator da inflação da variância (VIF) e índices de condição, não sendo observados valores indicativos de colinearidade (tolerância 0,90–0,97; VIF 1,02–1,11; maior índice de condição=14,95). Assim, todas as variáveis foram mantidas no modelo multivariado.

Não houve necessidade de submissão ao Comitê de Ética em Pesquisa por se tratar de estudo baseado em dados secundários. O SB Brasil 2023 foi previamente aprovado pela Comissão Nacional de Ética em Pesquisa — CONEP (Certificado de Apresentação para Apreciação Ética — CAAE nº 34497120.6.3001.0008; parecer nº 4.823.054).

### Declaração de disponibilidade de dados

Todo o conjunto de dados que sustenta os resultados deste estudo encontra-se disponibilizado no próprio artigo.

## RESULTADOS

A amostra incluiu 8.053 adolescentes, com idades entre 15 e 19 anos, dos quais 12,5% apresentaram o índice PUFA ≥1, ou seja, pelo menos um dente acometido por lesão de cárie não tratada, sendo a ocorrência mais comum o envolvimento pulpar (Tabela 1).

**Tabela 1:** Prevalência da cárie dentária não tratada e lesões bucais em dentes permanentes em adolescentes do Brasil, SB Brasil 2023.

Indicador	Presente			Ausente		
	%	IC95%	n	%	IC95%	n
**PUFA≥1**	12,5	10,1–15,2	1.076	87,5	84,8–89,9	6.965
**Envolvimento Pulpar**	9,9	7,9–12,3	848	90,1	87,7–92,1	7.193
**Ulceração**	2,2	1,7–3,0	251	97,8	97,0–98,3	7.790
**Fístula**	1,7	0,9–2,9	100	98,3	97,1–99,1	7.941
**Abscesso**	0,4	0,3–0,7	47	99,6	99,3–99,7	7.994

Percentuais calculados com base nos dados ponderados do plano amostral do SB Brasil, e o n reflete o valor bruto. PUFA ≥1: presença de, pelo menos, um dente permanente com lesão pulpar, fístula ou abscesso; IC95%: intervalo de confiança de 95%.

Ao considerar o índice PUFA ≥1 e as características demográficas da população avaliada, foram identificadas associações com algumas características socioeconômicas, tais como: cor/raça, baixa escolaridade, renda familiar e presença de benefícios de renda social, conforme detalhado na Tabela 2.

**Tabela 2:** Prevalência de PUFA ≥1 em dentes permanentes segundo características sociodemográficas em adolescentes do Brasil, SB Brasil 2023.

Variável	Categoria	Amostra % (n)	Prevalência PUFA ≥1 (%)	IC95%	F	Valor p
**Sexo**	Masculino	50,2 (3.871)	12,8	9,3–17,3	0,098	0,754
	Feminino	49,8 (4.170)	12,1	10,0–14,7		
**Idade (anos)**	15–16	43,2 (3.868)	12,7	10,1–15,9	0,225	0,792
	17–18	39,6 (2.749)	11,8	8,7–15,7		
	19	17,2 (1.424)	13,5	9,0–19,7		
**Cor/raça**	Branca	38,0 (2.600)	6,8	5,0–9,3	8,538	<0,001^*^
	Preta	12,5 (1.026)	18,4	13,1–25,3		
	Amarela	1,3 (107)	11,1	5,2–22,3		
	Parda	47,9 (4.199)	15,4	12,1–19,4		
	Indígena	0,4 (19)	27,9	5,3–72,9		
**Escolaridade**	Não estudou/EJA	0,5 (49)	42,2	16,0–73,7	10,957	<0,001^*^
	Fundamental	22,7 (2.000)	19,8	15,5–25,0		
	Médio	71,5 (5.431)	10,4	7,8–13,6		
	Superior	5,4 (479)	5,1	2,5–10,2		
**Renda familiar (SM)**	Até 1	30,7 (1.472)	18,6	13,5–25,1	19,017	<0,001^*^
	>1 a 3	49,0 (2.514)	13,5	10,5–17,2		
	>3	20,3 (1.025)	2,9	1,7–4,9		
**Recebe Bolsa Família**	Sim	32,9 (2.579)	20,4	15,4–26,4	24,782	<0,001^*^
	Não	67,1 (4.783)	8,6	6,7–11,0		
**Recebe BPC**	Sim	6,8 (518)	24,4	15,4–36,3	10,137	0,001^*^
	Não	93,2 (6.790)	11,6	9,3–14,2		
**Região**	Norte	10,8 (2.006)	19,4	14,9–24,9	2,240	0,094
	Nordeste	29,5 (2.859)	13,3	9,9–17,7		
	Sudeste	38,2 (919)	10,6	6,4–17,2		
	Sul	13,3 (1.088)	8,6	5,4–13,5		
	Centro-Oeste	8,3 (1.181)	15,0	12,2–18,4		

PUFA ≥1: presença de pelo menos um dente permanente com lesão pulpar, fístula ou abscesso; IC95%: intervalo de confiança de 95%; SM: saláriomínimo; BPC-LOAS: Benefício de Prestação Continuada. F ajustado: teste de independência com correção de Rao-Scott para amostras complexas. Amostra = número absoluto (não ponderado) de indivíduos por categoria;

*p<0,05: estatisticamente significativo.

O *Oral Impacts on Daily Performances* (OIDP) foi o instrumento utilizado para avaliar o impacto de problemas bucais na qualidade de vida dos adolescentes. Por meio desse instrumento, observou-se que, entre aqueles adolescentes cuja saúde bucal impactou a qualidade de vida, 22,4% apresentavam índice PUFA ≥1. Foram significativas as associações entre PUFA ≥1 e as variáveis relacionadas às atividades diárias, nas quais os adolescentes relataram: deixar de praticar esportes (50,5%), deixar de se divertir (43,0%), ter prejuízo nas atividades diárias (42,8%), dormir mal (35,9%), ficar nervoso/irritado (34,1%), dificuldades para falar (31,6%) ou comer (31,3%) e ter vergonha ao sorrir ou falar (21,6%), como destacado na Tabela 3.

**Tabela 3:** Associação entre impacto na qualidade de vida (OIDP) e presença de PUFA ≥1 em dentes permanentes de adolescentes brasileiros, SB Brasil 2023.

Variável (OIDP)	Categoria	PUFA≥1 (%)	PUFA=0 (%)	IC95% PUFA≥1	F ajustado	Valor p
**OIDP (algum impacto)**	Sim	22,4	77,6	17,7–27,9	66,596	<0,001^*^
	Não	6,4	93,6	4,9–8,3		
**Dificuldade para comer**	Sim	31,3	68,7	24,9–38,5	98,089	<0,001^*^
	Não	8,0	92,0	6,3–10,2		
**Dificuldade para falar**	Sim	31,6	68,4	19,1–47,5	17,116	<0,001^*^
	Não	11,2	88,8	9,2–13,6		
**Incomodado ao escovar**	Sim	14,1	85,9	8,7–22,2	0,385	0,535
	Não	12,1	87,9	9,8–14,9		
**Deixou de praticar esportes**	Sim	50,5	49,5	38,8–62,1	102,953	<0,001^*^
	Não	11,1	88,9	9,0–13,7		
**Ficou nervoso ou irritado**	Sim	34,1	65,9	26,1–43,1	87,938	<0,001^*^
	Não	8,3	91,7	6,7–10,1		
**Deixou de sair/se divertir**	Sim	43,0	57,0	31,5–55,3	71,680	<0,001^*^
	Não	10,3	89,7	8,3–12,7		
**Vergonha de sorrir/falar**	Sim	21,6	78,4	15,5–29,3	17,755	<0,001^*^
	Não	10,4	89,6	8,4–12,9		
**Atrapalhou atividades diárias**	Sim	42,8	57,2	28,0–59,1	36,819	<0,001^*^
	Não	10,3	89,7	8,5–12,6		
**Dormiu mal por problemas bucais**	Sim	35,9	64,1	28,6–43,9	155,151	<0,001^*^
	Não	7,3	92,7	5,9–9,2		

PUFA ≥1: presença de pelo menos um dente permanente com lesão pulpar, fístula ou abscesso; IC95%: intervalo de confiança de 95% (para PUFA ≥1); F ajustado: teste de independência com correção de Rao-Scott para amostras complexas;

*p<0,05: estatisticamente significativo; OIDP: *Oral Impacts on Daily Performances*.

A Tabela 4 mostra os agravos associados à presença do índice PUFA ≥1. Desse modo, pode-se destacar que 17,5% dos adolescentes identificaram a necessidade de tratamento por meio de uma autoavaliação. Já 79,5% dos adolescentes com PUFA ≥1 tiveram uma autopercepção de tratamento imediato de urgência, 30,5% desses adolescentes relataram dor nos últimos seis meses e 26,1% relataram perda de, pelo menos, um dente permanente.

**Tabela 4:** Associação entre agravos em saúde bucal e uso de serviços odontológicos com a presença de PUFA ≥1 em dentes permanentes em adolescentes do Brasil, SB Brasil 2023.

Variável	Categoria	PUFA≥1 (%)	PUFA =0 (%)	IC95% PUFA ≥1	F ajustado	Valor p
**Dor nos últimos 6 meses**	Sim	30,5	69,5	24,2–37,6	132,479	<0,001^*^
	Não	7,1	92,9	5,7–8,8		
**Autoavaliação de necessidade de tratamento**	Sim	17,5	82,5	14,4–21,2	79,171	<0,001^*^
	Não	2,8	97,2	1,7–4,4		
**Urgência percebida de tratamento**	Imediata	79,5	20,5	68,1–87,6	86,207	<0,001^*^
	Eletiva	14,8	85,2	11,3–19,2		
	Preventiva/Rotina	2,7	97,3	1,5–4,7		
	Sem necessidade	2,7	97,3	0,9–7,4		
**Perda de dentes permanentes (≥1)**	Sim	26,1	73,9	19,5–34,0	27,799	<0,001^*^
	Não	10,3	89,7	8,1–13,0		
**Oclusopatia**	Presente	16,0	84,0	12,6–20,1	5,124	0,024^*^
	Ausente	10,7	89,3	8,1–14,1		
**Sangramento gengival**	Presente	17,8	82,2	14,5–21,6	14,555	<0,001^*^
	Ausente	9,7	90,3	7,1–13,0		
**Presença de cálculo**	Presente	16,5	83,5	13,4–20,1	8,130	0,004^*^
	Ausente	10,2	89,8	7,5–13,8		
**Nunca foi ao dentista**	Nunca foi	19,1	80,9	12,6–27,9	5,185	0,023^*^
	Já foi	11,5	88,5	9,5–14,0		
**Última consulta no dentista (ano)**	≤1	11,2	88,8	8,9–14,1	0,205	0,651
	>1	12,0	88,0	9,3–15,3		
**Tipo de serviço procurado**	SUS	17,8	82,2	13,7–22,8	36,088	<0,001^*^
	Privado/Plano	6,0	94,0	4,5–8,0		
**Tipo de serviço na última consulta**	SUS	16,8	83,2	13,5–20,7	35,557	<0,001^*^
	Privado/Plano	6,7	93,3	5,1–8,8		

PUFA ≥1: presença de, pelo menos, um dente permanente com lesão pulpar, fístula ou abscesso; IC95%: intervalo de confiança de 95% (PUFA ≥1); SUS: Sistema Único de Saúde; F ajustado: teste de independência com correção de Rao-Scott para amostras complexas;

*p<0,05: estatisticamente significativo.

Pode-se também destacar que, dentre os adolescentes com PUFA ≥1, 19,1% nunca haviam comparecido a uma consulta odontológica. Contudo, 32,0% dos adolescentes que buscaram atendimento odontológico relataram odontalgia, e 17,7% dos adolescentes buscaram atendimento pelo SUS, conforme Tabela 4.

No modelo de regressão logística, observou-se que os principais fatores associados à presença de PUFA ≥1 foram as desigualdades socioeconômicas e a presença de agravos bucais. Após ajuste do modelo, adolescentes de cor/raça preta apresentaram 2,41 vezes mais chances de apresentar PUFA ≥1 em comparação aos brancos. Além disso, aqueles pertencentes a famílias com renda de até um salário-mínimo apresentaram 3,22 vezes maiores chances de PUFA ≥1 do que os adolescentes com renda superior a três salários-mínimos. A presença de dor (OR=3,54), o autorrelato de necessidade de tratamento (OR=3,59) e a perda dentária (OR=1,88) também foram fatores fortemente associados à presença de PUFA ≥1, após ajuste das chances, conforme Tabela 5.

**Tabela 5:** Modelo de regressão logística entre fatores sociodemográficos e agravos em saúde bucal com PUFA ≥1 em adolescentes do Brasil, SB Brasil 2023.

Variável	Categoria comparada	OR	IC95%	Valor p	OR ajustado	IC95% (ajustado)	Valor p
		Análise sem ajuste			Análise ajustada		
**Raça/cor**	Preto *vs*. Branco	3,07	1,85–5,09	<0,001^*^	2,41	1,27–4,56	0,030^*^
	Pardo *vs*. Branco	2,47	1,75–3,49		1,39	0,88–2,19	
**Escolaridade**	Fundamental Incompleto *vs*. Superior	4,24	1,92–9,40	<0,001^*^	2,30	0,78–6,71	0,005^*^
	Fundamental Completo *vs*. Superior	5,69	2,28–14,19		1,99	0,65–6,07	
	Médio *vs*. Superior	2,15	0,98–4,72		1,10	0,40–3,02	
**Renda familiar (SM)**	Até 1 *vs*. >3	7,68	3,97–14,85	<0,001^*^	3,22	1,69–6,14	0,007^*^
	1 a 3 vs. >3	5,23	2,89–9,45		3,08	1,68–5,67	
**Dor nos últimos 6 meses**	Com dor *vs*. Sem dor	5,75	4,16–7,94	<0,001^*^	3,54	2,47–5,08	<0,001^*^
**Autoavaliação necessidade de tratamento**	Sim *vs*. Não	7,44	4,48–12,37	<0,001^*^	3,59	1,94–6,66	<0,001^*^
**Perda dentária (≥1 dente)**	Sim *vs*. Não	3,07	1,99–4,74	<0,001^*^	1,88	1,06–3,33	0,031^*^

OR (*odds ratio*): razão de chances; IC95%: intervalo de confiança de 95%;

*p<0,05: estatisticamente significativo; SM: salário mínimo.

## DISCUSSÃO

O presente estudo avaliou dados de 8.053 adolescentes brasileiros, entre 15 e 19 anos, faixa etária em que mudanças físicas, emocionais e sociais tornam a aparência e a funcionalidade bucal relevantes para a autoestima e a qualidade de vida. Por se tratar de um estudo transversal, não é possível estabelecer a temporalidade das associações observadas, havendo risco de causalidade reversa. Assim, não se pode afirmar se as condições clínicas antecederam os impactos ou se estes influenciaram a percepção e o comportamento em saúde bucal. Assim, as associações encontradas devem ser interpretadas com cautela.

Os resultados destacaram a frequência de 12,5% dos adolescentes brasileiros entre 15 e 19 anos apresentando índice PUFA ≥1, ou seja, possuírem pelo menos um dente acometido por lesão de cárie não tratada, tendo como principal consequência o envolvimento pulpar (9,9%). De forma semelhante, Gomes *et al.* ^9^, ao avaliarem adolescentes de 12 anos, observaram que as consequências clínicas de lesões de cárie não tratadas (índice PUFA ≥1) ocorreram em 8,71% da amostra, com o comprometimento pulpar sendo o mais frequentemente também o comprometimento pulpar. Isso é particularmente preocupante em razão da gravidade das lesões cariosas e suas consequências, principalmente ao se considerar o pequeno intervalo (três anos) entre as idades dos adolescentes avaliados nos dois estudos, com aumento da frequência dos acometidos em quase quatro (3,99) pontos percentuais, considerando a faixa etária entre 15 e 16 anos (12,7%). Ainda, no presente trabalho, apesar de não significativo, houve aumento da prevalência do índice PUFA ≥1 entre as faixas etárias de 17–18 anos, passando de 11,8 para 13,5% aos 19 anos, com pior condição bucal nessa idade. O manejo dessa situação na saúde pública é complexo e demanda gestões intersetoriais dos serviços de saúde bucal, com planejamento, gerenciamento de recursos e maiores investimentos na atenção primária e especializada para identificar e tratar dentes com comprometimento pulpar^9^.

A prevenção da cárie dentária e o seu tratamento adequado extrapolam as dimensões funcional e estética dos dentes. A saúde bucal transcende o ambiente oral, influenciando a saúde geral e a qualidade de vida do indivíduo^11^. Ademais, as condições bucais durante a infância podem repercutir na idade adulta^12^. As consequências clínicas da cárie dentária não tratada foram associadas a problemas no sono de crianças em idade escolar^13^. Tal fato foi corroborado pelo presente estudo, no qual foi identificada uma associação estatisticamente significativa entre o índice PUFA ≥1 e a qualidade do sono (35,9%) dos adolescentes. Esses resultados enfatizam a importância do atendimento imediato perante as consequências da cárie dentária e representam um indicador crucial para o estabelecimento de prioridades nos serviços públicos de saúde e para a tomada de decisões clínicas^14^.

O tipo de alimento consumido também está relacionado à maior prevalência de cárie em idades precoces. Por meio de uma revisão sistemática, Cascaes *et al.* ^15^ observaram que a ingestão de alimentos ultraprocessados se associou à maior ocorrência de cárie dentária na infância e adolescência, evidenciando a necessidade de políticas públicas voltadas à modificação de determinantes sociais e à redução do consumo desses alimentos, visando à melhoria da saúde bucal de crianças e adolescentes^2,15,16^.

A distribuição desigual da cárie nas populações está fortemente associada à condição socioeconômica^17,18^. No presente estudo, características socioeconômicas, tais como cor/raça, escolaridade, renda familiar e recebimento de proventos decorrentes de benefícios sociais de renda foram estatisticamente associadas ao índice PUFA ≥1. Contudo, muitas variáveis têm sido consideradas preditoras de cárie dentária ao longo da adolescência. No SB Brasil de 2010, residir em cidades sem água fluoretada, com menor cobertura de abastecimento hídrico e em municípios com renda mediana mais baixa foram considerados fortes determinantes para a cárie dentária. Além disso, cor da pele, baixa renda familiar e presença de condições clínicas periodontais, como cálculo dentário ou sangramento gengival, foram associadas aos determinantes individuais de cárie dentária em crianças de 12 anos^5,17,19^.

Ao avaliarem a prevalência e gravidade de cárie em crianças brasileiras de 12 anos e sua associação com fatores individuais e contextuais, Freire *et al.* ^17^ identificaram que as crianças pretas e pardas apresentaram prevalência de cárie significantemente mais elevada que as crianças brancas. Contudo, os autores destacaram que essa diferença, em grande parte, foi devida à pior condição socioeconômica dos estratos de cor de pele preta e parda na população brasileira. Essas evidências podem ser extrapoladas para os resultados do presente estudo, uma vez que, além da população adolescente preta e parda, grande parte da população indígena também apresentou índice PUFA ≥1, sendo todas as populações possuidoras de baixas condições socioeconômicas. Adicionalmente, adolescentes em famílias com menor *status* socioeconômico, assim como os pertencentes a famílias com coesão familiar (união afetiva entre seus membros) de baixa a moderada, foram mais propensos a apresentarem maior número de lesões cariosas cavitadas^5,20^, corroborando os achados do presente estudo, em que o PUFA ≥1 foi três vezes mais prevalente nos indivíduos com renda abaixo de três salários-mínimos, mesmo após ajuste das variáveis.

Maiores níveis educacionais e de renda dos pais estão associados a maior atenção e cuidado com a saúde bucal dos filhos^21,22^. É possível que a escolaridade materna esteja associada à maior busca por serviços odontológicos entre adolescentes, visto que as escolhas parentais ainda exercem impacto nas decisões dos indivíduos nesse período^23^. Contudo, estudos avaliando a escolaridade dos adolescentes e o índice PUFA ainda permanecem escassos na literatura, indicando a importância dos achados desta pesquisa, uma vez que foi identificada associação estatisticamente significativa entre a menor escolaridade dos adolescentes brasileiros e o índice PUFA ≥1. As iniquidades persistem, com maior prevalência de cárie em grupos mais pobres, refletindo as marcantes desigualdades sociais no Brasil e seus impactos na saúde da população^17^.

Desse modo, evidencia-se a necessidade de uma política pública de saúde integral que articule família, escola e serviços de saúde. O escopo dos serviços deve ser cada vez mais ampliado, visto que as disparidades socioeconômicas ainda são um fator limitante no acesso aos serviços odontológicos. Além disso, o contexto em que os adolescentes estão inseridos também deve ser considerado. A conscientização sobre cuidados bucais precoces deve envolver a família e as escolas, para que sejam propostas estratégias visando reduzir o risco de doenças bucais na infância e adolescência. Adolescentes provenientes de ambiente escolar desfavorável (estudantes de escolas públicas e com maior índice de repetência escolar) apresentaram maior probabilidade de buscarem serviços odontológicos para tratamento e resolução dos sintomas^23^ de dor decorrentes da já deteriorada condição dentária, o que foi observado no presente trabalho, pela autopercepção de tratamento urgente imediato na grande maioria (79,5%) dos adolescentes com PUFA ≥1. Destaca-se aí a grande procura por parte desses adolescentes pelo atendimento via SUS.

A adolescência é um período de transição da infância para a idade adulta, que envolve mudanças psicológicas e fortes influências sociais, além de maior exposição a fatores de risco emocionais, sociais e físicos, tornando-se uma fase vulnerável a comportamentos nocivos e complexos^18,24^. Nesse contexto, o OIDP insere-se como um instrumento que mede a percepção dos indivíduos sobre o impacto social dos distúrbios orais em seu bem-estar. Diferentes estudos têm identificado correlações entre a presença de cárie e a redução na qualidade de vida de adolescentes por meio desse instrumento^1,25^. Tais achados corroboraram os identificados na presente pesquisa, uma vez que foi identificada uma associação entre a pior qualidade de vida e a presença de PUFA ≥1 em dentes permanentes de adolescentes brasileiros. Importante destacar alguns dos fatores associados à redução na qualidade de vida deles, uma vez que as consequências decorrentes da cárie não tratada impediram esses adolescentes de praticarem esportes (50,5%), se divertirem (43,0%) e realizarem suas atividades diárias (42,8%) e também afetarem o seu sono (35,9%). Tais evidências sobre o impacto da progressão da cárie na qualidade de vida de crianças e adolescentes subsidiam maior atenção dos formuladores de políticas públicas quanto à oferta de cuidados odontológicos baseados na prevenção, no diagnóstico precoce e no tratamento das lesões cariosas^2^.

Ademais, o índice PUFA permite rastrear doenças pulpares por meio de sinais clínicos intraorais; entretanto, o índice apresenta maior sensibilidade quando há cavitação perceptível. Já em casos de dentes restaurados, essa sensibilidade diagnóstica diminui^7^. Por se tratar de um processo crônico, a cárie dentária apresenta sinais e sintomas pulpares variáveis conforme a fase, aguda ou crônica, podendo um abscesso dentário tornar-se assintomático após a drenagem espontânea por fístula. Assim, uma única avaliação pode não refletir completamente a condição de uma criança com cárie dentária avançada^2^.

Os resultados da presente pesquisa destacaram que houve associação significativa entre o índice PUFA ≥1 e a presença de agravos à saúde dos adolescentes, tais como necessidade de atendimento de urgência, perdas de dentes permanentes, oclusopatias e doenças periodontais. Desse modo, adolescentes com índice PUFA ≥1 apresentaram maior probabilidade de necessitar de intervenções clínicas invasivas. Dentre esses adolescentes, 79,5% apresentaram uma autopercepção de necessidade imediata de tratamento odontológico em situações de urgência. Ferreira *et al.* ^26^ destacaram que percepções inadequadas de saúde bucal por parte dos jovens, ou a valorização insuficiente da saúde bucal podem aumentar a ocorrência de procura por um cirurgião-dentista apenas para a resolução dos sintomas, o que é corroborado pelo presente estudo. Lopes *et al.* ^23^ destacaram que a probabilidade de adolescentes com lesões cavitadas procurarem serviços odontológicos por causa da dor foi 9,0% maior do que a daqueles sem lesões cavitadas. Tal situação traz implicações para a saúde do indivíduo e impacta os serviços de saúde, que podem apresentar maior demanda por atendimentos de urgência.

Adicionalmente, foi identificada associação significativa entre a utilização de serviços odontológicos públicos (17,7%) e a presença de PUFA ≥1 em dentes permanentes em adolescentes brasileiros. Além disso, 32,0% dos adolescentes que buscaram atendimento odontológico relataram odontalgia, refletindo a progressão da cárie e a procura tardia por atendimento, já com envolvimento pulpar e dor. Isso reforça a importância da identificação adequada de grupos mais suscetíveis, a fim de direcionar e otimizar de forma mais eficaz as estratégias e os recursos de controle da doença^2,9^.

Hábitos de higiene oral devem ser incentivados para reduzir a incidência de cárie dentária^16^. Há um número crescente de fatores de risco que aumentam a probabilidade de desenvolvimento de problemas orais e cáries. No entanto, ainda não foi determinado quais fatores apresentam maiores riscos^18^. Assim, uma investigação sobre esses fatores de risco em adolescentes, particularmente em países em desenvolvimento, torna-se primordial, uma vez que os resultados podem variar dependendo das diferenças regionais^20^.

Além da renda familiar e da escolaridade, um dos principais fatores de risco para o desenvolvimento de agravos ao tecido pulpar decorrentes da cárie não tratada em adolescentes foi o fator cor/raça, de modo que a chance de adolescentes de cor/raça preta apresentarem PUFA ≥1 foi 2,41 vezes maior que a de adolescentes brancos, independentemente de sua escolaridade ou renda familiar. O mesmo ocorreu com os pardos (OR 1,39), ainda que em menor proporção. Contudo, Freire *et al.* ^17^ justificaram essa maior prevalência de cárie pela menor renda familiar dos pretos. Segundo Vazquez *et al.* ^18^, a distribuição da cárie nas populações está diretamente relacionada às condições socioeconômicas. Contudo, no presente estudo, foi identificado que, ao se incluírem no modelo de regressão logística a escolaridade e a renda como principais fatores socioeconômicos, a variável cor/raça permaneceu estatisticamente significativa para o PUFA ≥1.

As causas das desigualdades em saúde bucal permanecem inter-relacionadas e complexas. Intervenções precoces em saúde bucal podem influenciar positivamente a eficácia das ações, especialmente entre os grupos mais desfavorecidos^27^. A Estratégia Saúde da Família é fundamental para a sustentação da Vigilância em Saúde, ao garantir o acompanhamento do usuário desde o primeiro contato até a continuidade do cuidado, possibilitando maior conhecimento do território e do contexto social^28^.

Este estudo contribui para a prática do cirurgião-dentista na Atenção Básica ao fornecer evidências para a identificação de grupos vulneráveis e agravos bucais prioritários, qualificando o planejamento das ações no território. Ao reforçar a vigilância em saúde bucal, os achados subsidiam a organização da agenda, a estratificação de risco e o direcionamento de estratégias de prevenção e cuidado, além de apoiar gestores na definição de prioridades e na alocação racional de recursos, fortalecendo as ações de saúde bucal nos serviços públicos^8^.

Desse modo, políticas sociais direcionadas aos adolescentes de cor/raça preta, voltadas para a ampliação do acesso aos serviços odontológicos, tanto da atenção básica quanto da especializada, seja por meio de ações preventivas, seja por ações intervencionais, são fundamentais para a redução desses agravos em saúde bucal nessa população com maior vulnerabilidade.

## COMITÊ DE ÉTICA:

Não houve necessidade de submissão deste estudo ao Comitê de Ética em Pesquisa, uma vez que se trata de análise de dados secundários. Ressalta-se que o SB Brasil 2023 foi previamente aprovado pela Comissão Nacional de Ética em Pesquisa (Conep), em 3 de julho de 2021, sob o CAAE nº 34497120.6.3001.0008, com parecer nº 4.823.054.
